# Age‐Related Decreases in Colonic Neurokinin A Content and Release Monitored Using Electrochemical Immunosensors

**DOI:** 10.1111/nmo.70446

**Published:** 2026-09-23

**Authors:** Chloe Miller, Mark Yeoman, Bhavik Anil Patel

**Affiliations:** ^1^ School of Applied Sciences University of Brighton Brighton UK; ^2^ Centre for Lifelong Health University of Brighton Brighton UK

**Keywords:** 3D‐printed, immunosensor, intestinal tract, Neurokinin A

## Abstract

**Background:**

Aging increases the risk of bowel dysfunction and reduced quality of life. Although Neurokinin A (NKA)‐mediated smooth muscle contraction declines with age, it is unclear whether this reflects altered NKA content and/or release. This study examined age‐related changes in colonic NKA content and release and assessed whether changes correlated with fecal NKA.

**Methods:**

An electrochemical immunosensor was developed and characterized for NKA detection. Distal colon tissue and fecal pellets were collected from 5‐ and 22‐month‐old female C57BL6J mice. Basal and mechanically evoked tissue NKA release was recorded in the presence and absence of ondansetron. NKA content in tissue and fecal pellets were measured.

**Results:**

Sensor fabrication was validated by electrochemical and EDX analyses. The immunosensor detected NKA with a detection limit of 2.76 pM and high selectivity. Basal and mechanically evoked NKA release were significantly reduced with age. Ondansetron inhibited mechanically stimulated NKA release in 5‐month‐old but not 22‐month‐old colons. Tissue NKA content declined with age. The proportion of total NKA released decreased from 65% ± 22% in young tissue to 30% ± 23% in aged tissue. Fecal NKA levels were similarly reduced in older mice.

**Conclusions:**

Reduced NKA release with aging is associated with lower tissue content. The electrochemical immunosensor provides a simple, cost‐effective method for NKA quantification and could be adapted for other gastrointestinal neuropeptides. The decrease in fecal NKA levels supports further investigation of fecal NKA as a noninvasive marker of age‐related changes in bowel function.

## Key Points

- A sensitive and selective electrochemical immunosensor was developed for the quantification of Neurokinin A (NKA) in colonic tissue and fecal samples.
- Basal and mechanically evoked NKA release are significantly reduced in the aged mouse colon and are associated with lower colonic NKA content.
- Ondansetron inhibits mechanically stimulated NKA release in young but not aged colon, indicating age‐related alterations in serotonergic regulation of NKA signaling.
- Fecal NKA levels decline with age and mirror reductions in colonic NKA content and release, supporting further investigation of fecal NKA as a noninvasive biomarker of age‐related bowel dysfunction.

## Graphical abstract

A 3D‐printed electrochemical immunosensor revealed significant age‐related reductions in colonic Neurokinin A (NKA) content and release. Decreased fecal NKA levels mirrored tissue changes, highlighting the potential of NKA as a noninvasive biomarker of age‐related bowel dysfunction.

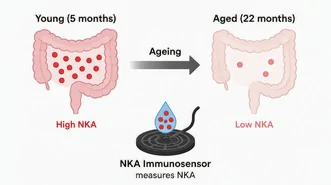

## 1 Introduction

The global population of adults over 80 years is increasing due to advances in medical research and healthcare that have extended life expectancy. However, improvements in longevity have not been matched by equivalent progress in managing age‐related gastrointestinal disorders. Bowel dysfunction is highly prevalent in older adults and commonly presents as chronic constipation and fecal impaction [1, 2]. These conditions contribute to significant morbidity, reduced functional independence, and diminished quality of life. A detailed understanding of the cellular and molecular changes that occur within the aging bowel is therefore essential for developing targeted interventions that improve outcomes in this vulnerable population.

Tachykinins are peptide neurotransmitters involved in regulating immune, gastrointestinal, and neuronal functions [3]. Within the enteric nervous system, neurokinin A (NKA), along with substance P and neurokinin B, are derived from the expression of preprotachykinin (PPT) genes in enteric neurons. Tachykinins exert their effects through NK1–NK3 receptors; NKA shows the highest affinity for the NK2 receptor, which is expressed on gastrointestinal smooth muscle cells and intrinsic nerve terminals [4]. Activation of NK2 receptors by NKA directly stimulates smooth–muscle contraction and modulates excitatory neural pathways, making NKA a key mediator of colonic motility and coordinated propulsive activity [5, 6]. As a result, altered tachykinin signaling may play a central role in the functional decline in motility observed with aging.

Previous work from our group has demonstrated intrinsic age‐related deficits in lower‐bowel neuromuscular function consistent with a chronic constipation phenotype. These studies showed a decrease in gut motility occurring with age‐related biomarkers. The studies linked age‐related decreases in colonic NKA to increasing bowel transit times. Exogenous NKA reduced transit time in 24‐month aged tissue, but was without effect in 3 months tissue, suggesting effective NKA signaling in the younger group. This shows the importance of NKA signaling and the potential for therapeutic targeting when reduced gut motility occurs [7, 8, 9]. Impaired NKA signaling, due to lower tissue content, has been shown in a range of GI diseases with reduced gut motility [10]. This suggests that with disease in the bowel, NKA molecule production is reduced, causing a decrease in motility. These studies therefore show promise for producing therapeutic targets via NKA receptors for gastrointestinal diseases and are useful in the literature to highlight the effects of NKA presence. However, these previous studies do not show quantifiable changes within the total content or of release of NKA, and specifically how these changes occur with age.

Quantification of neurokinin A (NKA) in bowel tissue typically relies on enzyme‐linked immunosorbent assays (ELISAs), liquid chromatography–mass spectrometry (LC–MS), or fluorescence‐based assays [11, 12, 13, 14]. These techniques provide sensitive and specific detection, with ELISAs capable of measuring NKA at sub‐picogram concentrations. LC–MS offers high analytical specificity and is commonly used in studies evaluating NKA metabolism or degradation. However, their application in GI research is limited by labor‐intensive protocols, extensive sample preparation, and the need for relatively large tissue volumes, making repeated or high‐throughput measurements impractical and costly. These limitations restrict their suitability for studying dynamic neuropeptide release or for assessing age‐related changes across multiple biological conditions.

This suggests there is a potential for a new technique to be used to quantify NKA within the GI tract in different age groups, using electrochemical sensors. Electrochemical biosensing offers a promising alternative for quantifying neuroactive molecules within the GI tract. Electrochemical sensors detect biomolecules through oxidation–reduction reactions and have been used extensively to measure electroactive neurochemicals [15, 16]. Electrochemical immunosensors combine the specificity of antibody‐based recognition with the simplified, low‐volume detection capabilities of electrochemical transduction, producing sensitive and selective assays that are more cost‐effective and time‐efficient than ELISAs or LC–MS [17, 18, 19]. We have previously shown such electrochemical immunosensors were able to detect changes in VIP and TNF‐α with age from fecal pellet samples [20, 21]. Their capacity for simplified analysis and compatibility with miniaturized platforms makes them particularly attractive for biomarker detection in aging research.

In this study, we developed a 3D‐printed patterned carbon black/polylactic acid (CB/PLA) electrochemical immunosensor capable of detecting NKA under physiologically relevant conditions. The sensors were assessed to measure over a biologically relevant concentration range (10–100 pM) and potential interferents. To the best of our knowledge, this represents the first application of an electrochemical immunosensor for direct NKA measurement in biological samples. By enabling simplified, sensitive, and low‐volume detection, this sensor addresses key limitations of existing methodologies and provides a scalable approach for monitoring neuropeptide signaling in the gut. Importantly, the development of a practical sensing tool for quantifying NKA lays the groundwork for future translational applications, including the identification of age‐related biomarkers, real‐time assessment of motility‐related dysfunction, and the potential development of diagnostic technologies to support clinical management of aging‐related gastrointestinal disorders.

## 2 Materials and Methods

### 2.1 Animals

All procedures were conducted according to UK Animals (Scientific Act), 1986 and associated guidelines and were approved by the University of Brighton Ethics Committee. Female C57BL/6J mice were obtained and housed in groups of 3–4 until required, at ages 5‐ and 22‐months‐old. Animals were maintained at 19.0°C ± 1°C, 55% humidity and fed on a maintenance diet (RM1 (E) 801,002 chow, Special Diet Services) and had free access to water.

The mice were euthanized then dissected to remove the distal colon. The distal colon was cut into 4 equal sized segments (2–3 mm length). Three pieces of tissue were utilized to investigate the release of NKA. The final piece of tissue was used to measure the total content of NKA within the tissue. Additionally, a formed fecal pellet in the distal region of the bowel was taken to measure the NKA levels.

### 2.2 Materials

Thiophene‐2‐carboxylic (Th2CA), 1‐ethyl‐3‐[3‐(dimethylamino)propyl]‐carbodiimide (EDC), N‐hydroxysuccinimide ester (NHS), potassium ferricyanide (III), potassium hexacyanoferrate (II) trihydrate, gold (III) chloride trihydrate, bovine serum albumin (BSA) were all purchased from Sigma. Phosphate‐buffered saline (PBS) pH 7.4 was prepared using 0.01 M disodium hydrogen phosphate, 0.01 M sodium dihydrogen phosphate, and 0.9% sodium chloride.

### 2.3 Fabrication of 3D‐Printed Patterned Neurokinin A Electrochemical Immunosensor

The 6 mm working electrode was fabricated using CB/PLA filament (ProtoPasta, USA). The knurl pattern of the electrode surface, as shown in Figure S1, was chosen as we have previously shown this increased the deposition of gold nanoparticles (AuNPs) [22]. The CB/PLA knurl patterned electrode was printed using a FlashForge Creator Pro 2 3D printer (FlashForge, CA, USA). The nozzle temperature was 210°C, the print bed temperature was 60°C, the layer thickness was 2 mm and print speed was 60 mm/s. These printing parameters were based on previous studies conducted [23, 24, 25]. For electrical connectivity, a silver wire was embedded in the working electrode and was then sealed using a glue gun. The CB/PLA knurl electrodes were electrochemically pretreated in 0.5 M NaOH at +1.2 V vs. Ag|AgCl for 200 s, followed by −1 V vs. Ag|AgCl for 200 s. To electrochemically deposit AuNPs onto the electrode surface, electrodes were placed in 5 mM HAuCl_4_ in 0.5 M H_2_SO_4_, where −0.5 V vs. Ag|AgCl was applied for 200 s. Following this, 1 mM of thiophene‐2‐carboxylic acid (Th2CA) monomer was dissolved in a 1:1 ratio of diethyl ether and triethyl ether and drop casted on the electrode and left to dry at room temperature. Th2CA was electropolymerized via cyclic voltammetry in 0.1 M PBS between 0 V and +1.6 V vs. Ag|AgCl for 3 cycles. The functional groups were activated through incubation of 10 mM EDC (1‐ethyl‐3‐(3‐dimethylaminopropyl)carbodiimide) and NHS (N‐hydroxysuccinimide) in 0.1 M PBS. These were then rinsed with 0.1 M PBS when activated. For the immobilization of Neurokinin A antibody, a rabbit polyclonal anti‐Neurokinin A antibody (Invitrogen PA5‐98181, Thermo Fisher Scientific), raised against recombinant human protachykinin‐1 (TAC1; amino acids 20–129), was used. The antibody has been validated by the manufacturer for ELISA, Western blotting, and immunohistochemistry applications. A 20 μL aliquot of antibody solution (100 μg/mL) was applied onto the activated Th2CA layer. This was left to dry on the surface to allow covalent bonds to occur between the carboxylic acid groups of the Th2CA and the amine groups of the antibody. To minimize the amount of nonspecific binding that could occur, the electrodes were blocked with 2% BSA for an hour.

### 2.4 Electrochemical Characterization of the NKA Immunosensor

Electrochemical measurements were conducted using a CHI760E potentiostat. A three‐electrode system was employed, consisting of an Ag|AgCl (3 M KCl) reference electrode, a platinum counter electrode, and a NKA immunosensor as the working electrode. To access the layer‐by‐layer fabrication of the immunosensor, cyclic voltammetry measurements were conducted in 5 mM potassium ferricyanide, and electrochemical impedance spectroscopy (EIS) measurements were conducted in 4 mM potassium ferri/ferrocyanide mixture solution in 1 M KCl at each layer.

The measurement protocol for the NKA sensor required measuring the difference in the current response before and after exposure in a sample. Initially, a control measurement was obtained in 5 mM potassium ferrocyanide using differential pulse voltammetry (DPV) at a potential window of 0–0.6 V vs. Ag|AgCl. The immunosensor was then placed in the sample for 2 h to allow for binding of any NKA present. Then an after response was recorded in 5 mM potassium ferrocyanide using DPV in the same potential window.

To investigate the optimal incubation time for an NKA‐antibody complex to be established, the NKA immunosensor was placed in 40 pM NKA at various time points ranging from 20 to 140 min. Calibration was conducted from 10 to 100 pM NKA. Selectivity studies were performed by incubating the NKA immunosensor for 2 h in solutions that contained molecules commonly found within the colon (serotonin, melatonin, norepinephrine, acetylcholine, ATP, Substance P, VIP, and Met‐Enkephalin), each at 400 pM. Potential interferents were evaluated at 400 pM, concentrations 10‐fold greater than the NKA concentration used in selectivity studies to provide a stringent assessment of sensor selectivity and evaluate potential nonspecific interactions under conditions where competing analytes were present in excess.

### 2.5 Electrochemical Measurement of Neurokinin A From Biological Samples

Prior to all biological measurements, the NKA immunosensors were run in 5 mM potassium ferricyanide using DPV to generate a control response.

For measurements of NKA release, three pieces of tissue were pinned into a 12 well plate lined with Sylgard containing oxygenated Kreb's buffer (95% oxygen, 5% carbon dioxide, pH 7.4; 117 mM NaCl, 4.7 mM KCl, 2.5 mM CaCl_2_, 1.2 mM MgCl_2_, 1.2 mM NaH_2_PO_4_, 25 mM NaHCO_3_, and 11 mM glucose) and 1X protease inhibitor cocktail (AEBSF—100 mM, Aprotinin—80 μM, Bestatin—5 mM, E64–1.5 mM, Leupeptin—2 mM, Pepstatin A—1 mM; ×100, Thermo Fisher Scientific). One tissue segment was pinned loosely and served as the basal control. Two additional tissue segments were manually stretched to the maximum extent possible without visible tissue damage and pinned under tension to provide mechanically stimulated responses. The same stretching procedure was applied to all preparations by a single investigator. Images of tissue preparations were acquired, and tissue surface area was quantified using ImageJ to assess the consistency of the mechanical stimulation protocol between age groups. The tissue was left for 20 min before the Krebs buffer was removed and replenished with 1 mL of warmed oxygenated Krebs buffer to commence the experiment. One of the mechanically stretched preparations was additionally incubated in 1 μM ondansetron, a 5‐HT_3_ receptor antagonist. Following 15 min incubation, a 20 μL aliquot was collected from each well and placed directly onto the surface of the NKA immunosensor and left for 2 h. The 15‐min collection interval was optimized in young tissue and subsequently applied uniformly to all age groups. While aging may influence NKA release kinetics, the use of a single collection interval across groups was considered important to avoid introducing age‐dependent sampling biases and to enable direct comparison of NKA release measurements. The immunosensor was then washed in 0.1 M PBS, and then a DPV measurement was conducted in 5 mM potassium ferricyanide. Post measurement, light microscopy images were taken of the tissue and analyzed using Image J to measure the surface area of the tissue to evaluate the degree of stretch.

To measure NKA content within a full thickness section of the colon, the tissue was homogenized in Kreb's buffer and centrifuged at 5000 rpm for 5 min. From this sample, a 20 μL aliquot of the supernatant was placed directly onto the surface of the NKA immunosensor and left for 2 h. The immunosensor was then washed in 0.1 M PBS, and a DPV measurement was conducted in 5 mM potassium ferricyanide. Results were normalized by the weight of the individual colonic tissue sample.

For the measurement of NKA levels within fecal pellets, one pellet was homogenized in 500 μL Kreb's buffer and centrifuged at 5000 rpm for 5 min. From this sample, a 20 μL aliquot of the supernatant was placed directly onto the surface of the NKA immunosensor and left for 2 h. The immunosensor was then washed in 0.1 M PBS and then a DPV measurement was conducted in 5 mM potassium ferricyanide. Results were normalized by the weight of the individual pellet sample.

### 2.6 Spike and Recovery Measurements

To assess matrix effects and recovery of NKA in biological samples, spike‐and‐recovery experiments were conducted using tissue homogenates and fecal pellet extracts. These were obtained from both age groups. A known concentration of NKA (40 pM) was added to each sample, and the resulting response was measured using the electrochemical immunosensor. Recovery was calculated by comparing the response obtained in the spiked sample to the expected response from the addition of 40 pM NKA.

### 2.7 Scanning Electron Microscopy (SEM)

SEM images were acquired using a Zeiss SIGMA field emission gun SEM, which was equipped with an Everhart–Thornley detector operating in secondary electron detection mode. The imaging was performed with a 5 kV accelerating voltage, a 20 μm aperture, and a working distance of 15 mm. The chemical composition at each step in the fabrication of the NKA immunosensor was determined using an Aztec energy dispersive X‐ray (EDX) spectroscopy system (Oxford Instruments), equipped with an X‐max 80 X‐ray detection. For SEM imaging and EDX spectroscopy mapping, the instrument was operated at a 20 kV accelerating voltage in a high vacuum.

### 2.8 Data Analysis

From cyclic voltammetry measurements, the anodic peak current and the difference between the anodic and cathodic peak potential (Δ*E*) were measured. From EIS measurements, the charge transfer resistance (Rct) was obtained following the fitting of experimental data in a modified Randles equivalent circuit. The release and content of NKA from both tissue and fecal pellets were normalized to the weight of the sample. The degree of stretch of the tissue for mechanically stimulated responses was obtained from measuring the tissue surface area using Image J. This was used to normalize the data where comparisons between mechanically evoked release in the presence and absence of ondansetron were made. Fractional release was calculated as the amount of NKA released under basal conditions during the 15‐min sampling interval divided by the total NKA content of the corresponding tissue sample and expressed as a percentage. This parameter was used to estimate the proportion of the available tissue NKA pool that is released and therefore provides an indication of release efficiency relative to peptide content. Statistical analyses were performed using GraphPad Prism 8.0. Data were assessed for normality using the Shapiro–Wilk test. Comparisons between two groups were analyzed using unpaired Student's *t*‐tests. Datasets involving multiple experimental conditions and age groups were analyzed using one‐way or two‐way ANOVA, as appropriate, followed by Tukey's or Sidak multiple comparisons post hoc test. Correlation analyses were performed using Spearman rank correlation coefficient. Data are presented as mean ± standard deviation (SD), and statistical significance was accepted at *p* < 0.05.

## 3 Results

**FIGURE 1:**
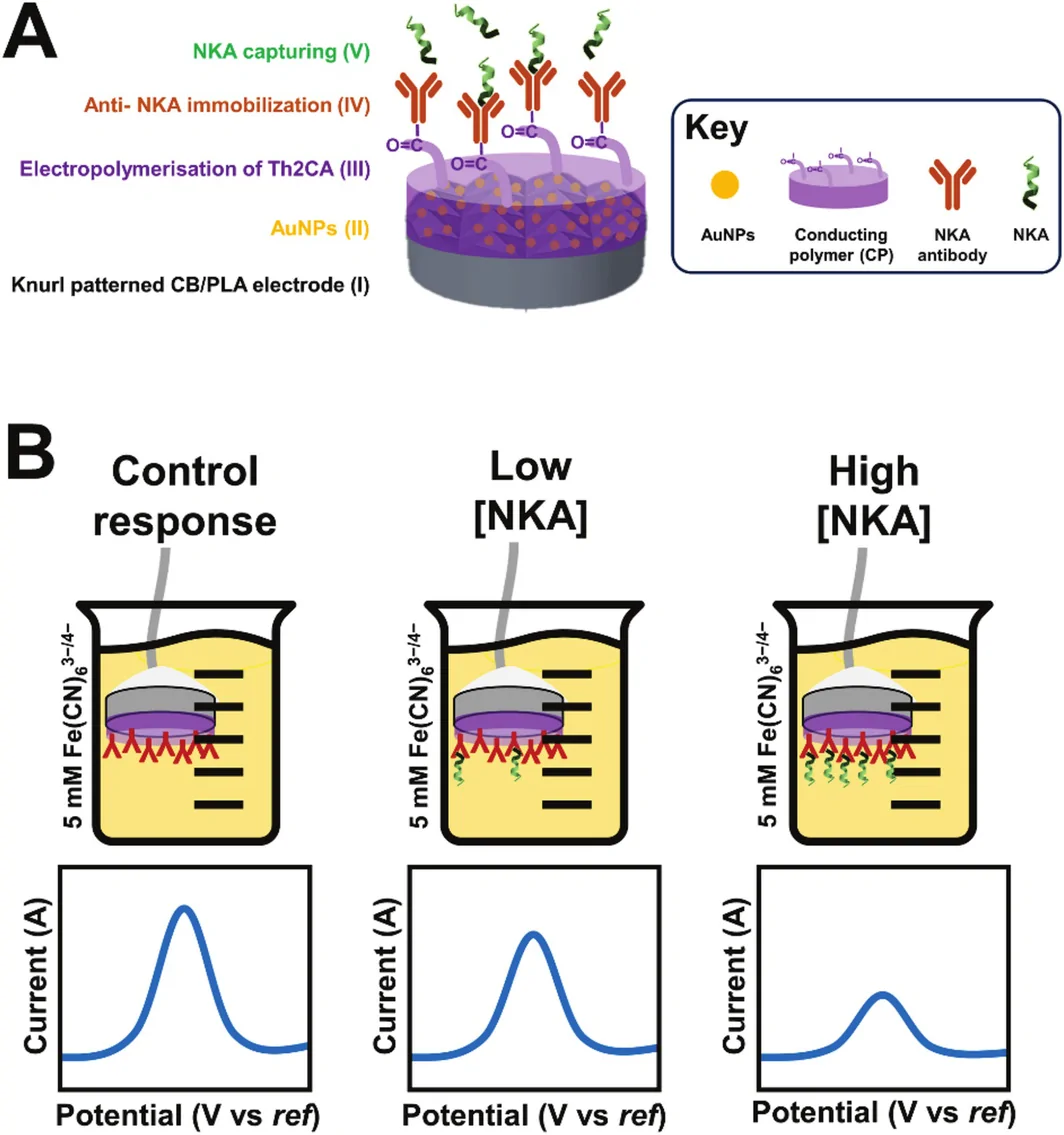
Schematic representation of the step‐by‐step fabrication of the NKA electrochemical immunosensor and how the immunosensor works in the presence of NKA molecules. (A) The 3D‐printed CB/PLA electrode was electrochemically pretreated (I) and AuNPs were drop casted on the CB/PLA electrodes (II). Thereafter, TH2CA was electropolymerized and carboxylic acid groups were activated (III). Next, anti‐NKA antibodies were covalently bonded to the activated Th2CA layer (IV). Finally, the electrode was ready to capture NKA molecules via the antigen–antibody binding step (V). (B) Various responses of the NKA sensor after incubation with or without NKA molecules to showcase the performance of the electrochemical immunosensor.

Figure 1A shows a schematic representation of the layers that form the developed NKA immunosensor. The base electrode is 3D‐printed using CB/PLA in a knurl pattern (Layer I). This layer was then deposited with gold nanoparticles (AuNPs; Layer II). Electropolymerization of the Th2CA conductive polymer, in which the carboxylic acid groups (Layer III) covalently bond to the amine group to immobilize the anti‐NKA antibody (Layer IV), ensures correct orientation on the antibody for the capture of NKA molecules. This ensures correct orientation on the antibody for the capture of NKA molecules.

Figure 1B indicates how the immunosensor works to detect NKA. When the NKA immunosensor is placed in potassium ferricyanide, a current signal is observed which acts as the control response. The electrode is then placed in a sample for 2 h, allowing NKA to bind the antibody. When a low amount of NKA binds to the antibody, this perturbs access to potassium ferricyanide and the current response decreases. Therefore, a reduction in the current is proportional to an increase in the amount of NKA within a biological sample.

**FIGURE 2:**
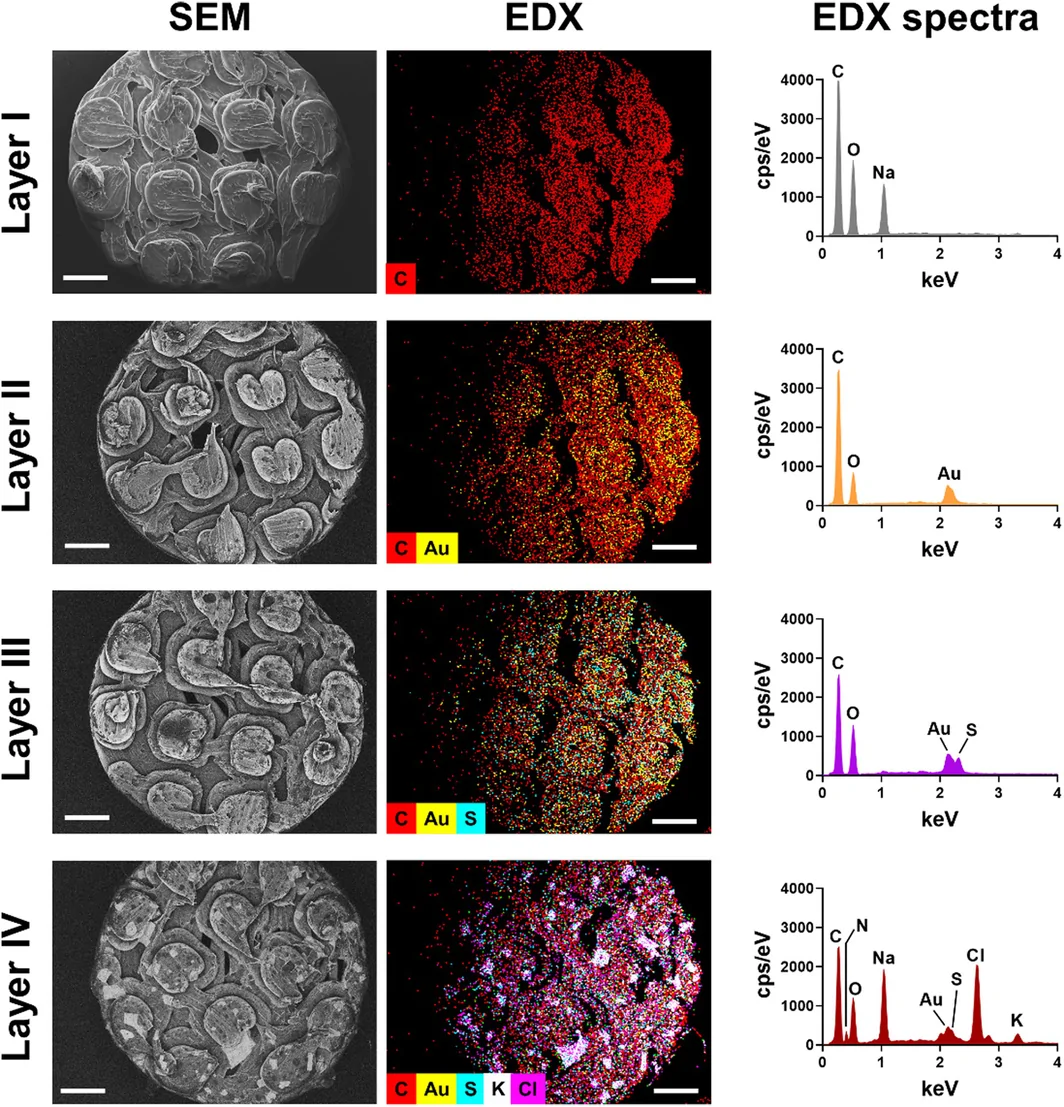
Characterization of the stages of immunosensor development on the surface of the patterned electrode via SEM and EDX analysis, where carbon (red), gold (yellow), sulfur (blue), potassium (K), and chloride (pink) are shown to be present on the different layers. Layer I SEM image Stage I, EDX analysis at Stage I show the distribution of carbon and EDX spectra at Stage I. Layer II SEM image at Stage II, EDX analysis at Stage II showing the distribution of carbon and gold and EDX spectra at Stage II. Layer III SEM image at Stage III, EDX analysis at Stage III showing the distribution of carbon, gold, and sulfur and EDX spectra at Stage III. Layer IV SEM image at Stage IV, EDX analysis at Stage IV showing the distribution of carbon, gold, sulfur, potassium, and chlorine and EDX spectra at Stage IV.

To verify the layer‐by‐layer production of the NKA immunosensor the chemical composition was assessed using SEM with EDX analysis and electrochemical measurements. Figure 2 shows SEM images and EDX analysis at each of the steps in the fabrication of the NKA immunosensor. The images and spectra in Layer I show the response of the bare CB/PLA electrode after electrochemical pretreatment. The knurl patterning of the electrode is clearly visible in the SEM image. The main chemicals observed on the electrode surface were carbon, oxygen, and sodium on the EDX spectra. The presence of sodium is due to the electrochemical pretreatment process which uses NaOH. Quantitative EDX analysis demonstrated that sodium was the predominant element present on the electrode surface (66.8 wt%), together with oxygen (24.2 wt%), chloride (4.6 wt%), and potassium (4.5 wt%). In Layer II, the AuNPs are deposited on the electrode, which is verified by the presence of a response on the EDX spectra. The EDX image shows even distribution of the Au on the electrode surface (Figure 2). This was further supported by quantitative EDX analysis, which showed gold accounted for 89.1 wt% of the detected elements on the electrode surface.

Within Layer III, the Th2CA conductive polymer was electropolymerized on the surface. Sulfur was observed on the EDX spectra, verifying the addition of the conducting polymer (Figure 2). Quantitative EDX analysis showed the presence of sulfur (18.0 wt%), together with gold (49.2 wt%) and oxygen (32.9 wt%), confirming successful deposition of the conducting polymer layer. In Layer IV there are clear changes on the SEM and EDX following the immobilization of the anti‐NKA antibody. Within the EDX spectra, the additional components included nitrogen, potassium, and chloride (Figure 2). Following antibody immobilization, quantitative EDX analysis showed clear changes in surface composition, with chlorine (37.6 wt%), sodium (30.6 wt%), oxygen (14.4 wt%), gold (10.3 wt%), potassium (6.3 wt%), nitrogen (1.8 wt%), and sulfur (0.8 wt%) detected on the electrode surface. The appearance of nitrogen provides further evidence of successful antibody immobilization due to the amine‐containing amino acids present within the antibody structure. The presence of the potassium and chloride ions comes from the formulation of the antibody solution that is drop‐cast onto the electrode. The SEM and EDX images clearly show the presence of each stage in the crafting of the NKA immunosensor.

To validate the SEM and EXD analysis data, we conducted electrochemical characterization of the electrode at each layer in the formation of the immunosensor. Cyclic voltammetry was carried using inner sphere redox probe potassium ferricyanide (Figure S2). There was a significant increase in the anodic current from layer I to layer II, followed by a significant decrease in the current in Layers III and IV (*p* < 0.001, *n* = 6, Figure S2B). The ΔE significantly decreased from Layer I to Layer II, followed by a significant increase in the Δ*E* in Layers III and IV (*p* < 0.001, *n* = 6, Figure S2C). Figure S2D displays the Nyquist plot obtained for each layer: CB/PLA electrode (I), AuNPs|CB/PLA (II), Th2CA|AuNPs|CB/PLA (III), and anti‐NKA‐Th2CA|AuNPs|CB/PLA (IV). Experimental data were fitted to a simplified equivalent Randle circuit to extract the Rct. The Rct significantly decreased from Layers I to II, followed by a significant increase in the Rct in Layers III and IV (*p* < 0.001, *n* = 6, Figure S2E). The increase in the current, reduction in Δ*E* and decrease in Rct from Layers I to II is expected as the deposition of AuNPs enhances the conductivity of the electrode [22]. The gradual decrease in the current, increase in Δ*E* and increase in Rct from Layers II to IV is expected as the layers increase the resistivity of the electrode. These findings also align with SEM and EDX results, further validating the layer‐by‐layer modification process.

To determine optimum incubation time for the binding of NKA on the immunosensor, a time dependent study was conducted. Figure S3A shows representative DPV traces taken after the immunosensor was placed in 40 pM NKA at times ranging from 20 to 140 min. Figure S3B shows the relationship between current and incubation time. The graph shows the current plateaus at 120 min indicating that the NKA is in equilibrium with the antibody on the sensor. These times are comparable to those used in commercially available ELISA assays that measure NKA [26].

**FIGURE 3:**
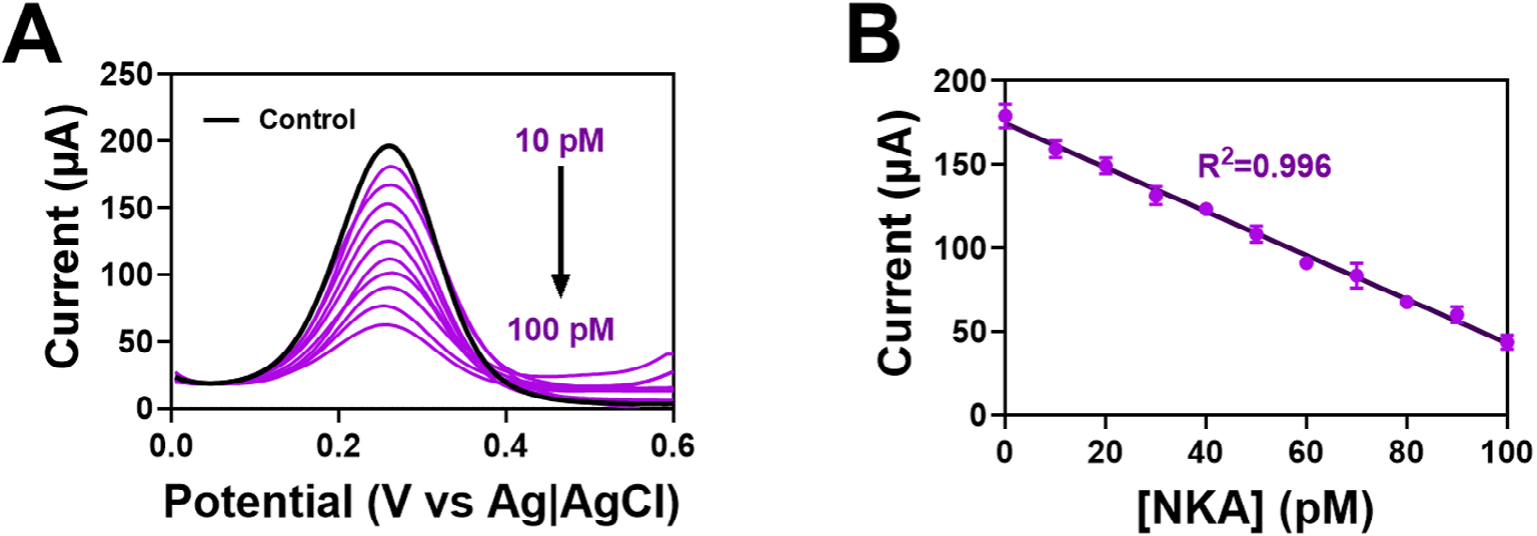
Calibration responses for the measurement of NKA. (A) DPV responses in 5 mM Fe (CN)_6_^3−^ following incubation of increasing concentrations of NKA from 10 to 100 pM. (B) Calibration response where the zero data point indicates the control response without NKA present. Data is shown as mean ± SD, *n* = 6.

For calibration studies, DPV responses in potassium ferricyanide were obtained after exposure of the immunosensor to varying concentrations of NKA (Figure 3A). The calibration responses are shown in Figure 3B. The developed immunosensor linear range was from 10 to 100 pM, with a correlation coefficient (*R* ^2^) of 0.996. The standard error of slope was 0.02 μA pM^−1^ and the standard error of intercept was 1.23 μA. The limit of detection was 2.76 pM (three times the error of the intercept).

To assess fabrication reproducibility, three independently fabricated batches of NKA immunosensors were evaluated (*n* = 6 per batch). The mean current responses to ferricyanide were 124.6 ± 2.2 μA, 123.3 ± 2.6 μA, and 123.0 ± 1.9 μA for Batches 1–3, respectively. The inter‐batch relative standard deviation was 0.7%, demonstrating excellent reproducibility of the sensor fabrication process.

**FIGURE 4:**
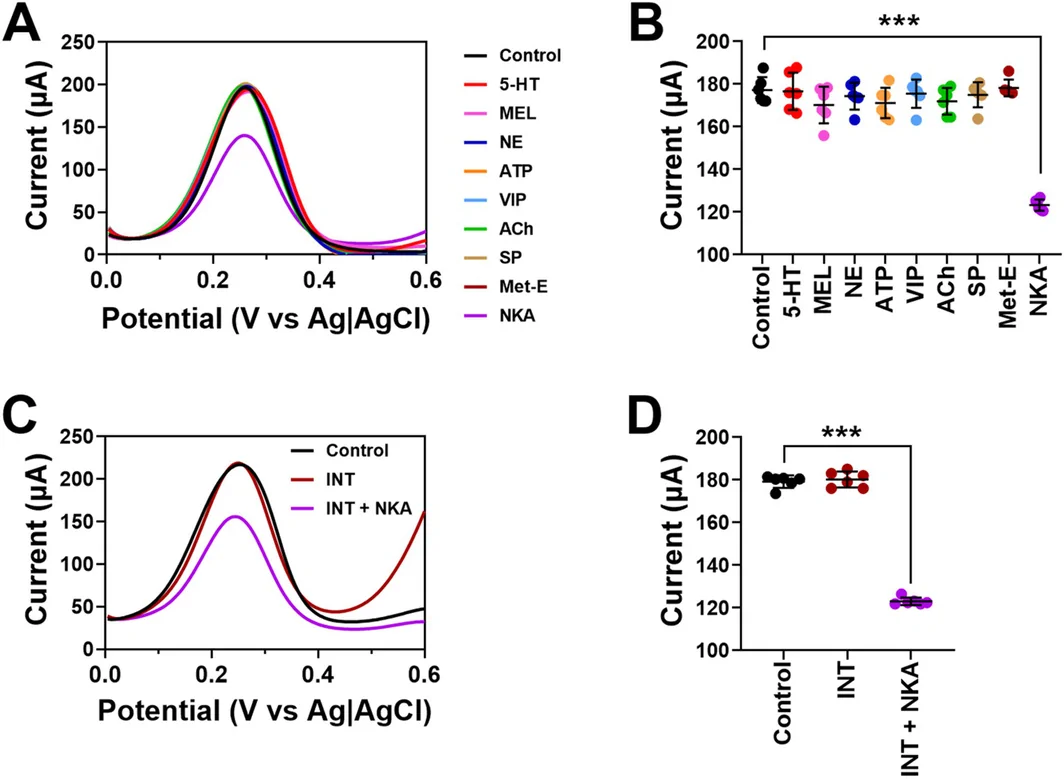
The current responses of potential interferences were studied using the NKA immunosensor. (A) DPV responses recorded in Fe(CN)_6_^3−^ after incubation with 400 pM serotonin, melatonin, norepinephrine, ATP, VIP, acetylcholine, Substance P, Met‐Enkephalin, in comparison to 40 pM of NKA. (B) Current responses. (C) DPV responses recorded in Fe(CN)_6_^3−^ after incubation with an interference mixture (INT) containing serotonin, melatonin, norepinephrine, ATP, VIP, acetylcholine, substance P, and Met‐enkephalin (all at 400 pM), and the same mixture supplemented with 40 pM NKA (INT + NKA). (D) Current responses. Data is shown as mean ± SD, *n* = 6, ***p < 0.001.

**FIGURE 5:**
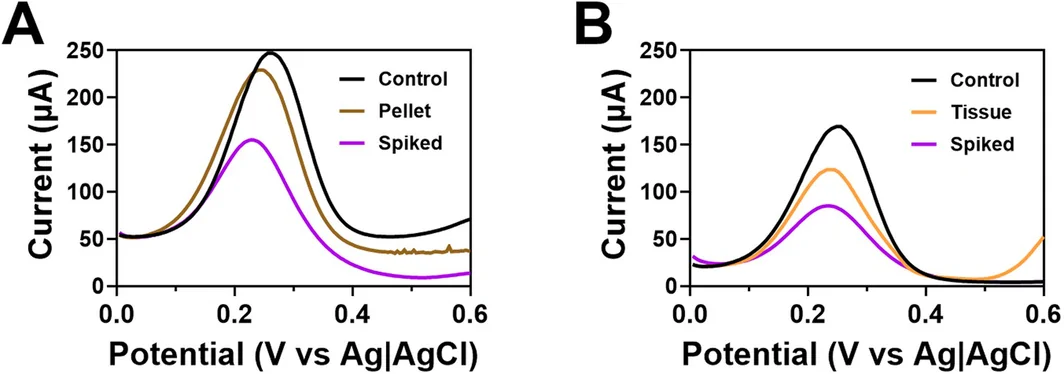
Assessment of matrix effects and recovery of NKA in biological samples. (A) Representative DPV responses recorded in potassium ferricyanide following incubation of the NKA immunosensor with fecal pellet extract (Pellet) and fecal pellet extract spiked with 40 pM NKA (Spiked), compared with the control response. (B) Representative DPV responses recorded in potassium ferricyanide following incubation with tissue homogenate (Tissue) and tissue homogenate spiked with 40 pM NKA (Spiked), compared with the control response.

To evaluate the selectivity of the NKA immunosensor, the sensor was incubated for 2 h with a range of interfering agents that are commonly found in the distal colon, and the DPV recorded in potassium ferricyanide as shown in Figure 4A. There was no significant difference in the response of the sensor from the control (Krebs buffered saline) and that observed following exposure to all the different biological interferents at 400 pM (Figure 4B). These findings indicate that these analytes do not alter the current response on the NKA electrochemical immunosensor through either interfering with the anti‐NKA antibody or directly influencing the response of potassium ferricyanide. Importantly, these potential interferents were tested at concentrations 10‐fold greater than the NKA concentration used in this study, demonstrating that the immunosensor maintains selective detection of NKA even when competing biomolecules are present in substantial excess. However, a significant decrease in the current was observed following exposure to 40 pM NKA when compared to the control measurement (*p* < 0.0001, *n* = 6, Figure 5B).

To further assess selectivity under more complex conditions, the immunosensor was exposed to an interference mixture (INT) containing serotonin, melatonin, norepinephrine, ATP, VIP, acetylcholine, substance P, and Met‐enkephalin, each at 400 pM (Figure 4C). No significant difference was observed between the control response and the response obtained in the presence of the interference mixture (Figure 4D). In contrast, supplementation of the interference mixture with 40 pM NKA (INT + NKA) resulted in a significant reduction in current (*p* < 0.0001, *n* = 6, Figure 4D). These findings demonstrate that the presence of multiple biologically relevant interferents does not compromise the selective detection of NKA by the immunosensor.

To assess potential matrix effects and recovery of NKA in biological samples, 40 pM NKA was analyzed in fecal pellet extracts and tissue homogenates. Representative DPV responses are shown in Figure 5. The addition of 40 pM NKA produced comparable reductions in current response in both biological matrices when compared with the control measurements. The mean current responses were 55.6 ± 7.5 μA, 55.2 ± 2.6 μA, and 55.3 ± 5.6 μA for buffer, tissue, and fecal matrices, respectively. The calculated recoveries were 99.3% for tissue homogenates and 99.5% for fecal pellet extracts, indicating minimal matrix interference and excellent analytical performance of the immunosensor in complex biological samples.

**FIGURE 6:**
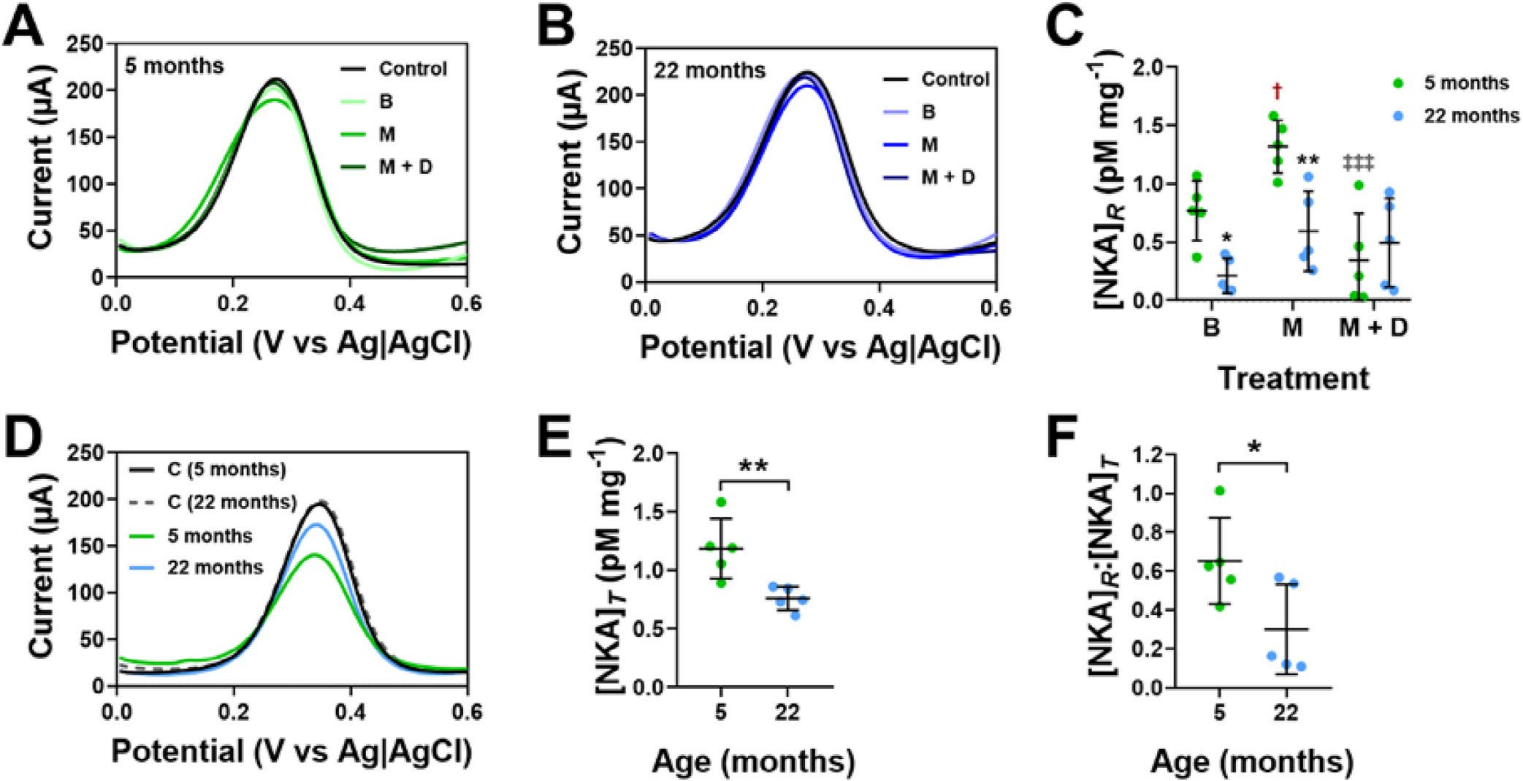
Murine distal colon release and total content studies to determine age‐related effects on the presence of NKA, where B is basal, M is mechanical and M + D is mechanical + drug. (A) Representative DPV responses of 5‐month‐old samples. (B) Representative DPV responses of 22‐month‐old samples. (C) The amount of NKA released following each treatment condition and in each age group, with all release values normalized to the individual weight of the colonic tissue. (D) Representative DPV reponses of NKA content from different age groups. (E) The amount of NKA content present within the different age groups, normalized to the individual weight of the colonic tissue. (F) Comparison of total NKA content and NKA release in the different age groups. Fractional release was calculated using the basal release value relative to the total NKA tissue content. Data is shown as mean ± SD, *n* = 5, *p < 0.05, **p < 0.01 shows an effect based on age, † based on mechanical effects, ‡ shows a drug effect based on the mechanical effects.

The release of NKA from colonic tissue was measured from 5‐ and 22‐month‐old female mice. Figure S4 shows that the supernatant obtained after 15 min of incubation in Krebs saline, from 5‐month‐old female mice, significantly reduced the current (*p* < 0.045, *n* = 3) and this did not vary significantly after 30 min when compared to 15 min (*p* = 0.841). This indicates that 15 min incubation is appropriate to accurately monitor the release of NKA from colonic tissue. The amount of NKA released after 15 min was measured from colonic tissue under basal (B) conditions, following mechanical stretch (M) and following mechanical stretch in the presence of 1 μM ondansetron (M + D). The image of the tissue preparation used for NKA release measurements is shown in Figure S5, and quantitative measurements of tissue surface area before and after mechanical stimulation are provided in Table S1. To assess the consistency of the mechanical stimulation protocol, tissue surface area was measured from tissue preparations. Mechanical stretch increased mean tissue surface area from 33.8 ± 12.8 mm^2^ to 57.6 ± 16.4 mm^2^ in 5‐month‐old tissue and from 25.5 ± 2.4 mm^2^ to 47.0 ± 12.3 mm^2^ in 22‐month‐old tissue (Table S1), indicating that a comparable degree of stretch was achieved between age groups. The current responses in potassium ferricyanide are shown after the NKA electrode was incubated for 2 h in the supernatant obtained from B, M and M + D samples from colonic tissue obtained from 5‐month‐old (Figure 6A) and 22‐month‐old animals (Figure 6B).

Under basal conditions, there was a significant reduction in NKA release from colonic tissue taken from 22‐month‐old animals compared to that from 5‐month‐old colon (*p* < 0.0244, *n* = 5, Figure 6C). When the colonic tissue from 5‐month‐old colon was mechanically stretched there was a significant increase in NKA release from basal to mechanical stretched (*p* < 0.0483, *n* = 5, Figure 6C), which was significantly reduced in the presence of ondansetron (*p* < 0.0007, *n* = 5, Figure 6C). However, for the 22‐month‐old animals, there was no difference in the release of NKA under basal conditions, when mechanically stretched or when mechanically stretched in the presence of ondansetron (Figure 6C). In stretched tissue NKA release in colonic tissue obtained from 22‐month‐old animals was reduced when compared to 5‐month‐old (*p* < 0.0029, *n* = 5, Figure 6C) although there was no difference in NKA release between the young and old stretched tissue in the presence of ondansetron (Figure 6C).

The current responses to potassium ferricyanide are shown after the NKA immunosensor was incubated for 2 h in the supernatant obtained from homogenized colonic tissue from 5‐ and 22‐month‐old animals (Figure 6D). There was a significant reduction in the total content of NKA present in colonic tissue obtained from 22‐month‐old animals when compared to 5‐month‐old (*t*(8) = 3.46, *p* < 0.0086, *n* = 5, Figure 6E). Fractional release was calculated as the amount of NKA released under basal conditions during the 15‐min sampling interval divided by the total NKA content of the corresponding tissue sample. This parameter reflects basal NKA release relative to tissue NKA content and does not represent the total releasable NKA pool. In 5‐month‐old animals, the fraction of release was 65% ± 22%, while this decreased significantly to 30% ± 23% in 22‐month‐old animals (*t*(8) = 2.47, *p* < 0.039, *n* = 5, Figure 6F).

**FIGURE 7:**
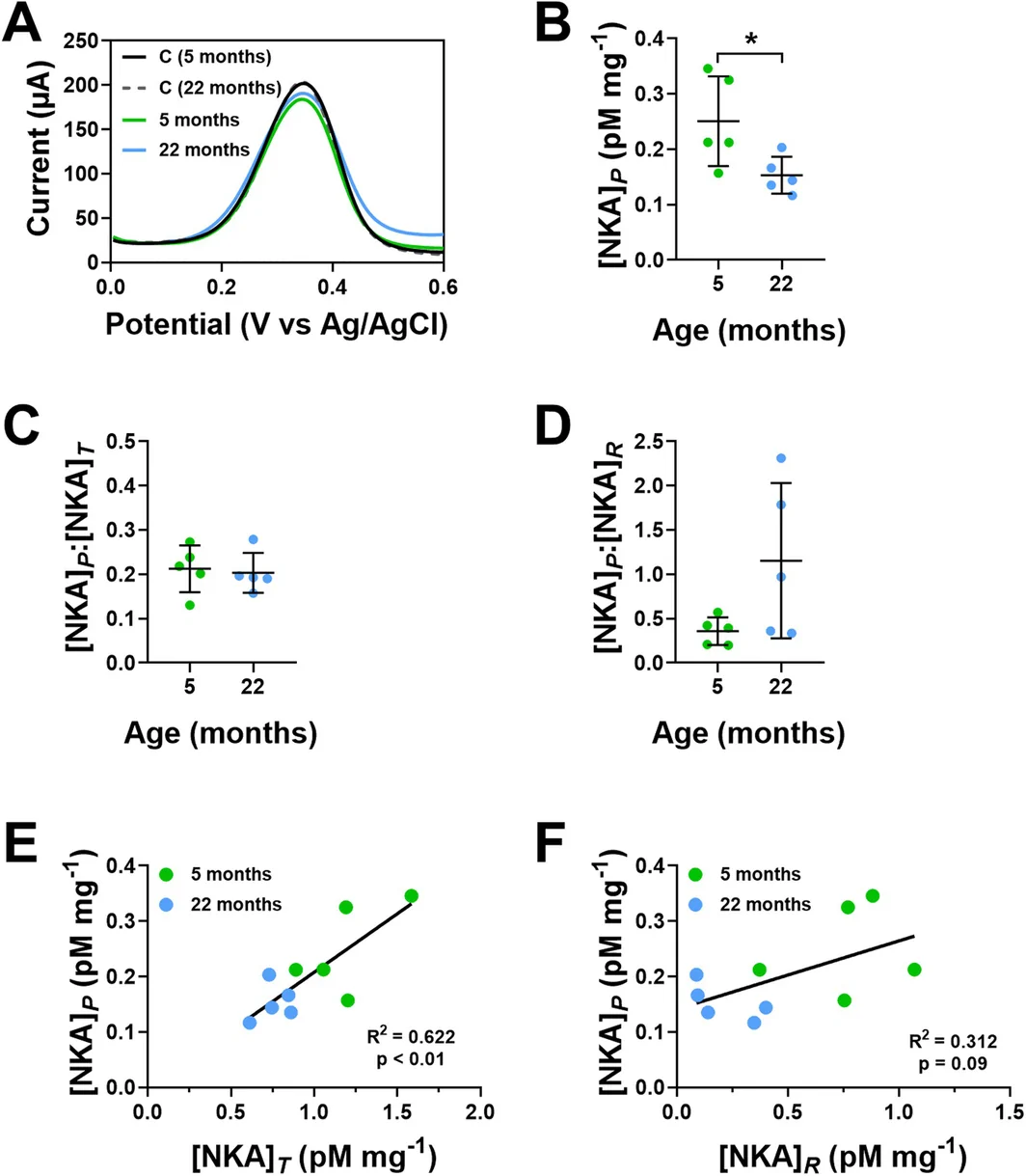
Fecal pellet analysis from 5‐ and 22‐month‐old murine distal colon samples. (A) Representative DPV responses after incubation on NKA immunosensors. (B) Comparison of NKA presence in fecal pellets, with the data normalized by the weight of the individual pellet. (C) Comparison of concentration of NKA from pellet to amount of NKA from content of colonic tissue for the different age groups. (D) Comparison of concentration of NKA from pellet to amount of NKA released from tissue for the different age groups. (E) Correlation of concentration of NKA from content of colonic tissue to NKA concentration in pellets for the different age groups. (F) Correlation of concentration of NKA released from colonic tissue to NKA concentration in pellets for the different age groups. Data is shown as mean ± SD, *n* = 5, *p < 0.05.

Finally, the current responses in potassium ferricyanide are shown after the NKA immunosensor was incubated for 2 h in the supernatant obtained from homogenized fecal pellets from 5‐ and 22‐month‐old animals (Figure 7A). There was a significant reduction in the amount of NKA present in fecal pellets obtained from 22‐month‐old animals when compared to 5‐month‐old pellets (*t*(8) = 2.49, *p* < 0.0378, *n* = 5, Figure 7B). When considering the amount of NKA present in the pellet, it represented approximately the same percent, 20%, of the content of NKA found in both 5 and 22‐month‐old colonic tissue (*n* = 5, Figure 7C). However, when the amount in the pellet was related to the amount released from the tissue, the 5‐month‐old pellet contained 32% of the NKA released; however, this increased to 71% of the NKA released from the 22‐month‐old tissue (*n* = 5, Figure 7D). Figure 7E shows that there was a significant correlation between the content of NKA in the pellet and the content present within colonic tissue (*p* < 0.0067, *n* = 10); however, there was no significant correlation between the amount of NKA in the pellet and the basal amount of NKA released from colonic tissue (*p* = 0.09, *n* = 10, Figure 7F).

## 4 Discussion

In this study, we fabricated an electrochemical immunosensor which was utilized to understand the effects of aging on the content and release of NKA from the distal colon. The fabricated NKA immunosensors had a low detection limit and a linear range that was suitable for physiological detection of NKA present in and released from a range of biological samples. The NKA immunosensor had high selectivity toward NKA in the presence of other biomolecules known to be widely present within the colon. Of note is the selectivity against substance P, which has a similar chemical structure. The current responses of the biomolecules known to be present within the colon mirrored the control response, indicating that there is no interaction between these biomolecules and the anti‐NKA antibody, nor was there any direct oxidation on the electrode surface. These findings strongly imply that the immunosensor is highly selective toward the detection of NKA. However, it should be noted that antibody specificity was assessed functionally through interference studies rather than by direct cross‐reactivity testing using purified tachykinins such as substance P or neurokinin B.

There are many current industrial methods that analyze the presence and concentration of tachykinins, such as ELISAs and FRET assays; however, these are considerably more time‐consuming, expensive, and have extensive sample preparation when compared to the NKA immunosensor. ELISAs can take up to 4 h before a measurement is taken [27], whereas immunosensors took 2 h in this study but can take a minimum of 45 min [21]. Utilizing 3D printing to craft the electrode that made the NKA immunosensors reduces the cost [28]. The use of potassium ferrocyanide as an indirect marker of NKA binding reduces the sample preparation required. Importantly, the strategy used to craft the NKA immunosensor can be tailored to monitor other important peptides or proteins. We have previously shown this approach to be effective in the detection of tumor necrosis factor alpha and vasoactive intestinal polypeptide from fecal pellets [20, 21]. This simple approach to make the immunosensor allows the development of sensing devices with the capability to measure multiple biomarkers and thus provides the potential to develop diagnostic and prognostic tools for a range of digestive diseases and disorders.

Aging decreased both basal and mechanically evoked release of NKA from colonic tissue. The source of the released NKA could be from both the mucosal epithelium and/or the enteric neurons. We have previously shown that NKA is co‐located with 5‐HT in colonic enterochromaffin (EC) cells [29]. In colonic tissue obtained from 5‐month‐old animals, there was a significant increase in NKA release following mechanical stretch when compared to basal. However, in colonic tissue from 22‐month‐old animals, no difference between basal and mechanically stretched tissue was observed. These findings demonstrate an age‐related impairment in mechanically evoked NKA release. Potential explanations include altered mechanosensory receptor function in EC cells, altered signaling through intrinsic primary afferent neurones (IPANs), ascending interneurons (AINs), or excitatory motor neurons (EMNs), or broader age‐related changes within enteric neural circuits.

Previous studies showed that aging is associated with attenuated high‐threshold afferent mechanosensitivity in the murine colon, and this was associated with a loss of TRPV1 channel function, which can reduce the activity of enteric neurons [30]. However, little is known about how mechanosensitivity changes with age in the EC cell. However, what is clear is that mechanically evoked 5‐HT release is still present with age from colonic EC cells [7, 9], but the overflow is increased due to a loss in SERT function [31].

Our previous studies demonstrated increased serotonin overflow in the aged murine colon [31, 32] and showed that aging reduces the force of contraction of the distal colonic mucosal reflex, the frequency and force of colonic migrating motor complexes (CMMCs) [8, 31, 33], and the NK_2_‐mediated component of both motility patterns. Furthermore, ondansetron, a 5‐HT_3_ receptor antagonist, inhibited components of these motility patterns in full‐thickness but not mucosa‐free preparations, supporting an important role for mucosal 5‐HT_3_‐dependent signaling in the regulation of colonic motor activity [9]. We therefore examined the impact of ondansetron on mechanically evoked NKA release. In colonic tissue obtained from 5‐month‐old animals, ondansetron significantly reduced mechanically evoked NKA release, whereas no significant effect was observed in tissue from 22‐month‐old animals. Taken together, these findings provide evidence that aging disrupts serotonergic regulation of NKA release within the colon. While the precise site of dysfunction cannot be identified from the present experiments, the loss of ondansetron sensitivity in aged tissue is consistent with impairment somewhere within the mucosal 5‐HT/5‐HT_3_ receptor pathway that links mechanical stimulation to NKA release. Potential explanations include altered 5‐HT_3_ receptor function or expression, altered EC‐cell mechanosensory signaling, or downstream changes within enteric neural circuits.

Additionally, we also observed a reduction in the total level of NKA within colonic tissue. Using super‐resolution fluorescence microscopy, we have previously demonstrated that the NKA peptide and 5‐HT are colocalized in the large dense core vesicles in the basolateral EC cell extensions [29]. However, we have also previously shown that 5‐HT content within the colonic mucosal epithelium does not differ between 3‐ and 24‐month‐old animals [31, 32]. If the age‐related reduction in NKA content originates from the mucosal compartment, these observations suggest that it is unlikely to simply reflect a reduction in EC‐cell number. Instead, the reduction in NKA may reflect alterations in peptide synthesis, storage, packaging, turnover or release.

Our previous studies also demonstrated an age‐related reduction in myenteric neuronal 5‐HT content [31]. Therefore, the observed decrease in NKA content could additionally reflect changes within enteric neuronal populations. While our previous work suggests that overall myenteric neuronal number is largely maintained with age, the present data cannot exclude age‐related changes in neuronal phenotype, neuropeptide expression, neuronal connectivity, or peptide handling.

We obtained the fraction of NKA released by obtaining the ratio of the basal NKA released during the 15‐min sampling interval and total content of NKA. We observed approximately a 35% reduction in the fraction of NKA released with increasing age, indicating that aging affects not only the total amount of NKA present within the tissue but also the efficiency with which the available NKA pool is released. This observation raises the possibility that aging alters additional processes regulating NKA availability or release.

Our previous studies demonstrated that NK_2_ receptor‐mediated contractions evoked by a range of stimuli are reduced with increasing age [7, 9]. We also showed that this reduction did not appear to result from altered NK_2_ receptor responsiveness, as contractions evoked by exogenous application of NKA were not different between young and old tissue. Taken together, these findings are consistent with the hypothesis that age‐related reductions in NKA content and release contribute to the impaired tachykinin‐dependent motor responses observed in aged colon. However, because contractility and NKA measurements were not assessed simultaneously in the present study, a direct causal relationship cannot be established.

Given our findings that NKA content and release decreased with increasing age, NKA may have potential as a biomarker for age‐related bowel dysfunction. However, measurement of tissue content or release would require an invasive approach involving tissue biopsies. Therefore, we investigated whether the age‐related changes observed in colonic NKA content and release were also reflected in NKA content of fecal samples.

We observed an age‐related decrease in fecal pellet NKA, consistent with the reductions observed in colonic tissue content and release. The NKA present within fecal pellets may originate from NKA released into the lumen or from NKA‐containing EC cells that are shed into the gut lumen as part of normal epithelial turnover [34]. The amount of NKA present in fecal pellets represented a similar proportion of the total tissue NKA content in both young and aged animals, suggesting a lack of age‐related changes in luminal processes responsible for the degradation of peptides.

However, this relationship was not observed when fecal NKA levels were compared with tissue NKA release. Despite greater variability, a significantly larger proportion of fecal NKA relative to tissue release was observed in the aged animals. This interpretation was further supported by the observation that fecal NKA levels correlated with tissue NKA content but not with basal NKA release.

These findings suggest that fecal NKA may more closely reflect tissue NKA content than ongoing NKA release. While the cellular origin of fecal NKA cannot be determined from the present study, the data are consistent with contributions from tissue‐associated NKA stores, including shed epithelial cells.

Collectively, these findings support exploration of fecal NKA as a noninvasive marker of colonic NKA content. However, because fecal NKA did not significantly correlate with NKA release, the present data do not support its use as a direct surrogate measure of NKA release or enteric neurotransmission. Additional studies will be required to establish whether fecal NKA can predict functional changes in bowel motility during aging.

Several limitations of this study should be acknowledged. First, only female mice were examined. Female animals were selected to maintain experimental consistency and reduce biological variability associated with sex as an additional experimental factor. Furthermore, age‐related bowel dysfunction and constipation are particularly prevalent in older females [35, 36], making this a clinically relevant model. However, gastrointestinal motility, serotonergic signaling, and neuropeptide expression exhibit sex‐dependent differences [37, 38]. Therefore, the present findings should be interpreted within the context of female animals and may not be directly translatable to males.

Second, although aging was associated with reduced NKA content and release, the precise cellular and molecular mechanisms underlying these changes were not directly investigated. The present study cannot distinguish between potential contributions from altered tachykinin synthesis, peptide storage, enteric neuronal phenotype, neuronal connectivity, release mechanisms, or receptor‐mediated regulation. Furthermore, while mechanical stimulation was standardized using tissue surface area measurements, stretch was applied manually and therefore minor variability in the force applied between preparations cannot be excluded.

Third, while significant age‐related effects were detected, the study was conducted using modest sample sizes consistent with previous investigations of age‐related gastrointestinal dysfunction [7, 20, 31, 33]. Consequently, the correlation analyses should be considered exploratory, and larger studies will be required to confirm the relationships observed between fecal and colonic NKA and to determine whether fecal NKA predicts tissue NKA independently of age.

Overall, the present study demonstrates age‐related reductions in colonic NKA content and release. Basal NKA release, mechanically evoked NKA release, tissue NKA content, and fecal NKA levels were all significantly reduced in aged animals, accompanied by a loss of ondansetron sensitivity. Together, these findings indicate that aging alters both NKA availability and release within the colon. These observations are consistent with previous reports of impaired NK_2_‐dependent colonic motor function in aged mice [7, 9]. The detection of NKA in fecal matter highlights its potential as a noninvasive marker of age‐related changes in colonic NKA biology; however, further studies are required to establish its utility as a biomarker of bowel dysfunction.

## 5 Conclusions

We created a simple and cheap approach to craft electrochemical immunosensors which can be easily tailored for the detection of different peptides and proteins in various gastrointestinal samples. Using this approach, we developed a highly sensitive and selective electrochemical immunosensor for the detection of NKA. We were able to detect NKA release from colonic tissue, stored in colonic tissue and present within fecal pellets. We observed an age‐related decrease in NKA release and content from colonic tissue. In young animals, mechanically stretched colonic tissue released a higher amount of NKA, which was blocked in the presence of the 5‐HT_3_ antagonist ondansetron. However, in older animals, no increases in NKA release were observed when the colonic tissue was mechanically stretched. Finally, age‐related changes in NKA levels were observed in fecal pellets, highlighting the potential of fecal NKA as a noninvasive biomarker of age‐related bowel dysfunction.

## Author Contributions

**Chloe Miller:** conceptualization, data curation, formal analysis, investigation, validation, writing – original draft and review and editing. **Mark Yeoman:** conceptualization, visualization, methodology, supervision, writing – review and editing. **Bhavik Anil Patel:** conceptualization, visualization, methodology, formal analysis, project administration, writing – review and editing.

## Conflicts of Interest

The authors declare no conflicts of interest.

## Supporting information


**Figure S1:** The CB/PLA electrode. (A) Computer‐aided design (CAD) of the knurl patterned electrode and (B) light microscopy images of the CB/PLA printed electrodes with the knurl pattern. Scale bar is 2 mm.
**Figure S2:** Electrochemical characterization of the Neurokinin A immunosensor at each stage of production; (I) Electrochemically pretreated carbon base material layer, (II) AuNPs layer, (III) Th2CA conducting polymer layer, and (IV) Antineurokinin A antibody layer. (A) Representative cyclic voltammograms of each layer of the immunosensor, conducted in 5 mM Fe (CN)_6_^3−^ after incubation. (B) Current responses of each layer of the immunosensor. (C) ΔEp values of each layer of the immunosensor. (D) EIS responses of each layer of the immunosensor 5 mM Fe (CN)_6_^3−/4−^. (E) Rct values from the EIS spectra of each layer. Data is shown as mean ± SD, *n* = 6, *n* = 3 for Rct values, *p < 0.05, **p < 0.01, and *** *p* < 0.001.
**Figure S3:** Incubation studies to determine optimal incubation time for a stable electrochemical response to occur in the presence of NKA. (A) DPV responses at various time points from 20 to 140 min in the presence of 40 pM NKA. (B) Relationship between current and time responses. Data is shown as mean ± SD, *n* = 4.
**Figure S4:** Incubation times of tissue in Kreb's buffer after initial stabilization. (A) Representative DPV conducted in 5 mM Fe (CN)_6_^3−^ after 15 and 30 min. (B) Relationship between current and time taken for incubation. The control is antibody response only, without the presence of NKA molecules. Data is shown as mean ± SD, *n* = 3, *p < 0.05.
**Figure S5:** Representative microscopy images of pinning the distal colon for the release studies based on the conditions set. B is basal, M is mechanical, M + D is mechanical + drug (Ondansetron).
**Table S1:** Quantification of tissue surface area under basal and mechanically stretched conditions. Tissue surface area was measured from images using ImageJ. Data are presented as mean ± SD (*n* = 5 for basal and *n* = 10 for mechanical). Mechanical stretch increased tissue surface area in both 5‐ and 22‐month‐old colonic preparations, indicating a comparable degree of stretch between age groups.

## Data Availability

The data that support the findings of this study are available from the corresponding author upon reasonable request.
