# Supplementary material for: Age‐Related Decreases in Colonic Neurokinin A Content and Release Monitored Using Electrochemical Immunosensors
Source: Neurogastroenterol Motil. 2026 Sep 23;38(9):e70446. doi: 10.1111/nmo.70446 (PMC13601819; doi:10.1111/nmo.70446)
Supplement: Supplementary file 1 [file NMO-38-e70446-s001.docx]

# **Supporting Information**

# Age-related decreases in colonic Neurokinin A content and release monitored using electrochemical immunosensors

Chloe Miller ^1,2^, Mark Yeoman ^1,2^, Bhavik Anil Patel ^1,2*^

^1^School of Applied Sciences and ^2^Centre for Lifelong Health, University of Brighton, Brighton, UK, BN2 4GJ.

*Corresponding author: Bhavik Anil Patel; School of Applied Sciences and Centre for Lifelong Health, University of Brighton, Brighton, BN2 4GJ, UK; Telephone: +44 1273 642418 Email: b.a.patel@brighton.ac.uk

Figure S1. The CB/PLA electrode. (A) Computer aided design (CAD) of the knurl patterned electrode and (B) light microscopy images of the CB/PLA printed electrodes with the knurl pattern. Scale bar is 2 mm.

Figure S2. Electrochemical characterisation of the Neurokinin A immunosensor at each stage of production; (I) Electrochemically pretreated carbon base material layer, (II) AuNPs layer, (III) Th2CA Conducting Polymer layer, (IV) Anti Neurokinin A antibody layer. (A) Representative cyclic voltammograms of each layer of the immunosensor, conducted in 5 mM Fe (CN)_6_^3−^ after incubation. (B) Current responses of each layer of the immunosensor. (C) ΔEp values of each layer of the immunosensor. (D) EIS responses of each layer of the immunosensor 5 mM Fe (CN)_6_^3−/4−^ . (E) Rct values from the EIS spectra of each layer. Data is shown as mean ± SD, n = 6, n =3 for Rct values, * p < 0.05, ** p < 0.01 and *** p <0.001.

Figure S3. Incubation studies to determine optimal incubation time for a stable electrochemical response to occur in the presence of NKA. (A) DPV responses at various time points from 20 – 140 minutes in the presence of 40 pM NKA. (B) Relationship between current and time responses Data is shown as mean ± SD, n = 4.

Figure S4. Incubation times of tissue in Kreb’s buffer after initial stabilization. (A) Representative DPV conducted in 5 mM Fe (CN)_6_^3−^ after 15 and 30 minutes. (B) Relationship between current and time taken for incubation. The control is antibody response only, without the presence of NKA molecules. Data is shown as mean ± SD, n = 3, * p < 0.05.

Figure S5. Representative microscopy images of pinning the distal colon for the release studies based on the conditions set. B is basal, M is mechanical, M + D is mechanical + drug (Ondansetron).

*Table S1. Quantification of tissue surface area under basal and mechanically stretched conditions. Tissue surface area was measured from images using ImageJ. Data are presented as mean ± SD (n = 5 for basal and n=10 for mechanical). Mechanical stretch increased tissue surface area in both 5- and 22-month-old colonic preparations, indicating a comparable degree of stretch between age groups.*

| **Age group** | **Basal area (mm^2^)** | **Stretched area (mm^2^)** | **% increase** |
| --- | --- | --- | --- |
| **5 months** | 33.8 ± 12.8 | 57.6 ± 16.4 | 70.4 |
| **22 months** | 25.5 ± 2.4 | 47.0 ± 12.3 | 84.5 |
